# Predicting in-hospital mortality after cardiac surgery using machine learning: temporal validation in the MIMIC-IV database

**DOI:** 10.3389/fcvm.2026.1914633

**Published:** 2026-08-31

**Authors:** Jianhui Zhou, Chengxin Zhang

**Affiliations:** Department of Cardiovascular Surgery, The First Affiliated Hospital of Anhui Medical University, Hefei, Anhui, China

**Keywords:** cardiac surgery, logistic regression, machine learning, MIMIC-IV, mortality prediction, risk stratification, SHAP, temporal validation

## Abstract

**Background:**

Machine learning (ML) models have been increasingly applied to surgical risk prediction; however, their comparative performance and temporal generalizability remain inadequately evaluated.

**Methods:**

A retrospective cohort study of 9,956 adult patients undergoing coronary artery bypass grafting and/or valve surgery was conducted using the MIMIC-IV v3.1 database. Logistic regression (LR), random forest (RF), and XGBoost models were developed without class weighting and tuned within nested cross-validation, trained on patients from 2008 to 2016 and temporally validated on patients from 2017 to 2019. Demographics, comorbidities, and 13 laboratory values available within 24 h of admission served as predictors. A class-weighted variant with isotonic recalibration was examined as a sensitivity analysis.

**Results:**

In-hospital mortality was 2.1% in both the training and validation sets. In temporal validation, LR achieved the highest AUC (0.876), followed by RF (0.856) and XGBoost (0.831); the paired difference vs. XGBoost was +0.044 [95% CI (0.002, 0.084)], marginally excluding zero, whereas the difference vs. RF was not significant. Without class weighting, all three models were well calibrated (calibration intercepts near zero). Age, blood urea nitrogen, creatinine, and partial thromboplastin time were the most influential predictors.

**Conclusions:**

Under a fair comparison with tuned, unweighted models, logistic regression achieved the highest numerical discrimination, but the advantage was small and at most marginally significant. Logistic regression provides an interpretable and well-calibrated model for early-admission mortality risk stratification, whereas these data do not establish that tree-based models are intrinsically less temporally stable.

## 1 Introduction

Cardiac surgery, including coronary artery bypass grafting (CABG) and valve procedures, carries a perioperative mortality risk of 1%–5% (1–3). Accurate perioperative risk stratification informs clinical decision-making and quality benchmarking. Traditional risk scores—EuroSCORE II and the STS score—were developed using logistic regression and may exhibit declining calibration in contemporary practice (4–7).

Machine learning (ML) has been proposed to improve risk prediction by capturing nonlinear relationships (8, 9). Recent cardiac surgery studies have applied random forest and gradient boosting with mixed results (10–12), and systematic reviews suggest that ML often does not outperform logistic regression, particularly when event rates are low (13, 14).

A frequently overlooked aspect is temporal generalizability—models trained on historical data may degrade when applied to newer cohorts owing to shifts in practice and demographics (15, 16). Temporal validation, splitting data by calendar period, provides a more rigorous assessment than random partitioning, yet remains uncommon in cardiac surgery ML studies (17).

Using the MIMIC-IV database, which spans over a decade of surgical care (18), this study aimed to (1) develop and compare LR, RF, and XGBoost models for predicting in-hospital mortality after cardiac surgery, (2) evaluate temporal stability by training on 2008–2016 and validating on 2017–2019 data, and (3) identify key predictors using SHAP analysis.

## 2 Materials and methods

### 2.1 Data source and study population

This retrospective cohort study used MIMIC-IV v3.1, a de-identified database of patients admitted to Beth Israel Deaconess Medical Center between 2008 and 2019 (18). The institutional review boards of the Massachusetts Institute of Technology and Beth Israel Deaconess Medical Center approved data collection; individual consent was waived for de-identified data. The analysis and reporting followed the TRIPOD + AI statement (19); the completed checklist is provided as Supplementary Material.

**Figure 1:**
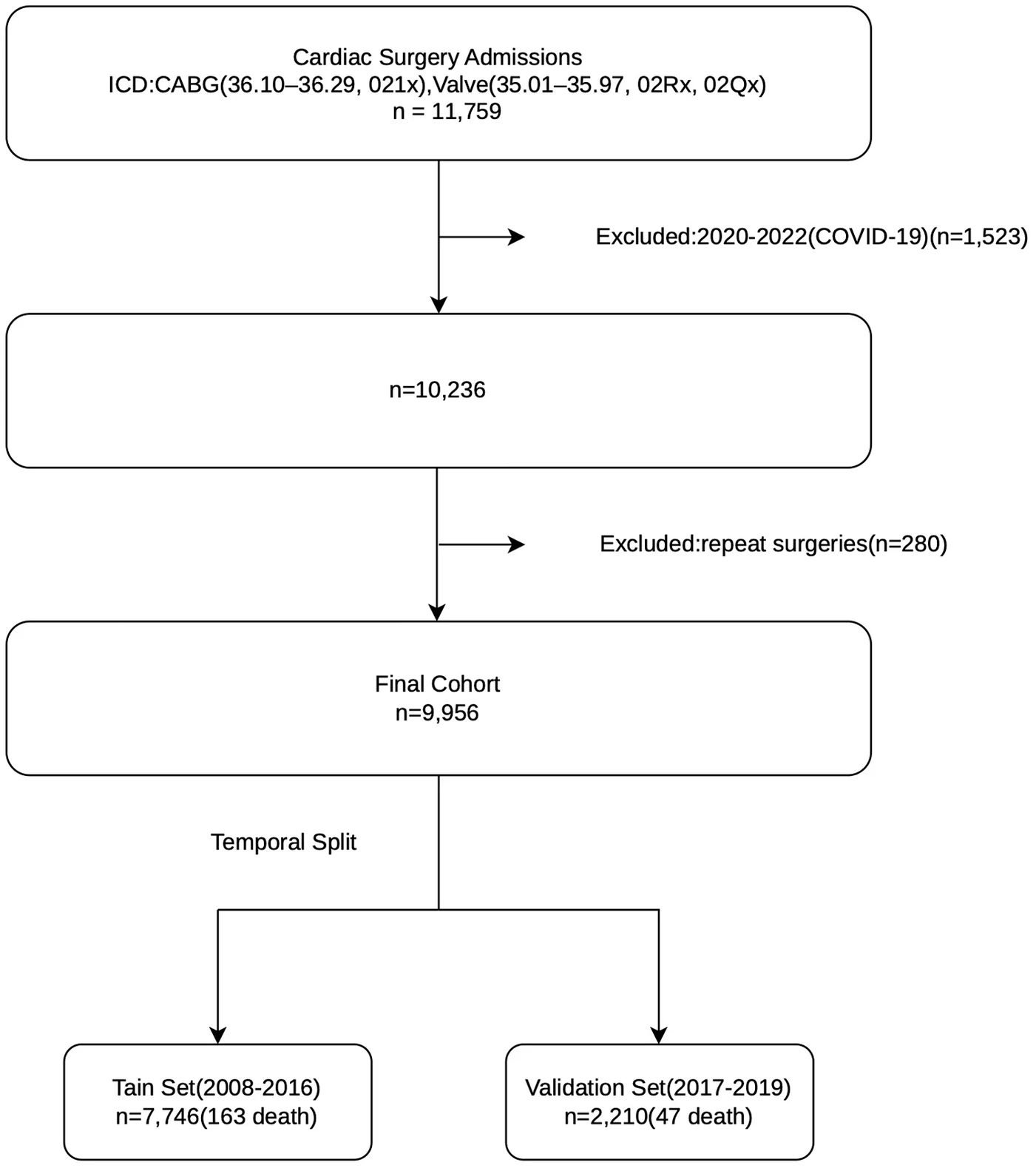
Patient selection flowchart. CABG, coronary artery bypass grafting; Valve, valve surgery; ICD, international classification of diseases.

Adult patients (≥18 years) undergoing cardiac surgery were identified using International Classification of Diseases (ICD) procedure codes: CABG (ICD-9-CM 3610–3629; ICD-10-PCS 021 series) and valve surgery (ICD-9-CM 3501–3597; ICD-10-PCS 02R, 02Q series). For patients with multiple cardiac surgery admissions, only the first was retained. Patients from 2020 to 2022 were excluded owing to COVID-19-related confounding. In total, 11,759 cardiac surgery admissions were identified; after excluding the 2020–2022 period (leaving 10,236 admissions) and retaining only the first cardiac surgery per patient, 9,956 patients remained (7,746 training, 2008–2016; 2,210 validation, 2017–2019). The patient selection process is illustrated in Figure 1.

### 2.2 Outcome and predictors

The primary outcome was in-hospital mortality. Predictors included: (1) demographics—age and sex; (2) admission type—elective, urgent, or emergent; (3) surgery type—isolated CABG, isolated valve, or combined CABG + valve; (4) nine comorbidities derived from ICD codes—hypertension, diabetes mellitus, chronic kidney disease (CKD), chronic obstructive pulmonary disease (COPD), coronary artery disease (CAD), cerebrovascular disease, peripheral vascular disease, liver disease, and obesity; and (5) 13 laboratory values obtained within 24 h of admission—creatinine, hemoglobin, white blood cell (WBC) count, platelet count, sodium, potassium, glucose, blood urea nitrogen (BUN), albumin, total bilirubin, international normalized ratio (INR), partial thromboplastin time (PTT), and troponin T. The model is intended for early-admission risk stratification; the unweighted logistic model yields calibrated individual mortality probabilities, whereas weighted variants are used only for ranking.

### 2.3 Temporal split and model development

Patients were divided by calendar period: training set (anchor_year_group 2008–2010, 2011–2013, and 2014–2016) and temporal validation set (2017–2019). All continuous features were standardized to zero mean and unit variance with parameters derived exclusively from the training set.

Three models were developed without class weighting: (1) logistic regression with L2 regularization; (2) random forest; and (3) XGBoost. Hyperparameters were selected by an inner 5-fold stratified cross-validation maximizing the ROC AUC, repeated within every training fold (nested resampling). Search spaces were: logistic regression C ∈ {0.01, 0.1, 1.0, 10.0}; random forest max_depth ∈ {5, 10, None}, min_samples_leaf ∈ {1, 5}, max_features ∈ {sqrt, log2}; XGBoost learning_rate ∈ {0.01, 0.05, 0.1}, max_depth ∈ {3, 5, 7}, subsample ∈ {0.8, 1.0}. Median imputation and standardization were performed within each training fold. As a sensitivity analysis, a class-weighted variant was retained and isotonically recalibrated using development-set out-of-fold predictions only, with calibration reported before and after recalibration. Internal performance was evaluated using 5-fold stratified cross-validation on the training set. Models were then retrained on the full training set and evaluated once on the temporal validation set. SHAP values were computed for the XGBoost model (20). No variable selection was performed; all 28 predictors were included in each model to ensure fair comparison across modeling approaches with identical feature sets. All analyses were performed using Python 3.11 (21) with scikit-learn 1.9 (22), XGBoost 3.2, and SHAP 0.51.

### 2.4 Statistical analysis

Continuous variables were expressed as mean ± standard deviation and compared using the independent t-test. Categorical variables were expressed as frequency (percentage) and compared using the chi-squared test. Model discrimination was assessed using the area under the receiver operating characteristic curve (AUC) and area under the precision-recall curve (PR-AUC). Calibration was evaluated using the Brier score (31) and calibration plots. Calibration intercept and slope were estimated by fitting a logistic regression of the observed outcome on the logit of the predicted probabilities (logit[observed] = intercept + slope × logit[predicted]); perfect calibration corresponds to intercept 0 and slope 1 (23). Model superiority was assessed with paired-bootstrap 95% confidence intervals for the difference in AUC and PR-AUC computed on the same validation cases. Coefficients of the unweighted logistic model are reported with bootstrap 95% confidence intervals (1,000 resamples, repeating imputation and scaling within each resample); *p*-values are not reported because valid inferential standard errors are not available from a penalized predictive model. All performance metrics are reported as means with 95% confidence intervals from 5-fold cross-validation. A two-sided *p* < 0.05 was considered statistically significant.

Missing laboratory data were handled using median imputation, with imputation parameters derived exclusively from the training set. Among the 13 laboratory variables, missing rates ranged from 4% (creatinine) to 84% (troponin T); detailed missing rates and complete laboratory values for all variables are presented in Supplementary Table S1. Median imputation was adopted for several reasons. First, the low event rate (2.1%, 163 events in the training set) means that the asymptotic properties on which multiple imputation relies may be limited when the number of events is small relative to the number of predictors. Second, multiple imputation assumes that data are missing at random (MAR), an assumption that cannot be verified empirically and may be tenuous for laboratory tests ordered selectively on clinical indication (e.g., troponin T, 84% missing); although high missingness alone does not establish that MAR is violated, it precludes confident reliance on imputation-model assumptions. Third, median imputation is a transparent approach that preserves the central tendency of each variable without introducing distributional assumptions. This choice is supported by evidence that simple imputation methods perform comparably to multiple imputation in cardiovascular prediction models when the most influential predictors are not missing (24). However, median imputation may attenuate variance and weaken associations between predictors and outcome, particularly for variables with high missingness, and this limitation is further addressed in the Discussion.

## 3 Results

### 3.1 Cohort characteristics

After applying inclusion criteria, 9,956 patients were analyzed: 7,746 in the training set (2008–2016) and 2,210 in the temporal validation set (2017–2019) (Table 1). The mean age was 68.8 ± 12.4 years in the training set and 68.7 ± 11.4 years in the validation set. The proportion of female patients was 31.9% and 28.0%, respectively. Emergent admissions accounted for 21.2% of training cases and 8.2% of validation cases. In-hospital mortality was 2.1% (163/7,746) in the training set and 2.1% (47/2,210) in the validation set.

**Table 1:** Baseline characteristics of the training and temporal validation cohorts.

Characteristic	Training (2008–2016)	Validation (2017–2019)	*p*-value
	(*n* = 7,746)	(*n* = 2,210)	
Age, years	68.8 ± 12.4	68.7 ± 11.4	0.673
Female, *n* (%)	2,472 (31.9)	618 (28.0)	<0.001
Surgery type			
Isolated CABG	3,588 (46.3)	1,163 (52.6)	<0.001
Isolated valve	2,975 (38.4)	768 (34.8)	
CABG + Valve	1,183 (15.3)	279 (12.6)	
Admission type			
Elective	3,635 (46.9)	1,054 (47.7)	<0.001
Urgent	2,471 (31.9)	974 (44.1)	
Emergent	1,640 (21.2)	182 (8.2)	
Comorbidities			
Hypertension	6,043 (78.0)	1,801 (81.5)	<0.001
Diabetes mellitus	2,688 (34.7)	829 (37.5)	0.016
CKD	1,480 (19.1)	466 (21.1)	0.041
COPD	1,446 (18.7)	380 (17.2)	0.122
CAD	5,944 (76.7)	1,774 (80.3)	<0.001
Cerebrovascular disease	826 (10.7)	202 (9.1)	0.042
Peripheral vascular disease	1,467 (18.9)	383 (17.3)	0.092
Liver disease	260 (3.4)	135 (6.1)	<0.001
Obesity	1,097 (14.2)	432 (19.5)	<0.001
Laboratory values			
Creatinine, mg/dL	1.2 ± 1.1	1.1 ± 1.0	0.153
Hemoglobin, g/dL	12.2 ± 2.1	12.3 ± 2.0	0.004
WBC count, ×10⁹/L	10.2 ± 5.8	10.1 ± 4.7	0.559
Platelets, ×10⁹/L	188.6 ± 72.7	182.2 ± 69.8	<0.001
Sodium, mmol/L	139.2 ± 3.2	139.6 ± 3.3	<0.001
Potassium, mmol/L	4.2 ± 0.5	4.3 ± 0.5	<0.001
Glucose, mg/dL	135.4 ± 50.4	136.3 ± 55.8	0.506
BUN, mg/dL	21.4 ± 13.0	20.0 ± 11.4	<0.001
Albumin, g/dL	3.9 ± 0.5	3.9 ± 0.5	0.101
Total bilirubin, mg/dL	0.7 ± 0.6	0.6 ± 0.4	0.380
INR	1.3 ± 0.4	1.3 ± 0.4	0.408
PTT, seconds	39.5 ± 23.9	41.5 ± 27.5	0.002
Troponin T, ng/mL	0.8 ± 1.9	0.8 ± 1.7	0.676
In-hospital mortality, *n* (%)	163 (2.1)	47 (2.1)	1.000

Values are presented as mean ± standard deviation or *n* (%). CABG, coronary artery bypass grafting; Valve, valve surgery; CKD, chronic kidney disease; COPD, chronic obstructive pulmonary disease; CAD, coronary artery disease; WBC, white blood cell count; BUN, blood urea nitrogen; INR, international normalized ratio; PTT, partial thromboplastin time. Laboratory values reflect available measurements within 24 h of admission; representative missing rates: creatinine 4%, hemoglobin 4%, WBC 5%, platelets 5%, BUN 4%, albumin 64%, total bilirubin 59%, troponin T 84%. Complete laboratory data for all 13 variables are available in Supplementary Table S1. *p*-values were calculated using the t-test for continuous variables and the chi-squared test for categorical variables.

### 3.2 Model performance

**Figure 2:**
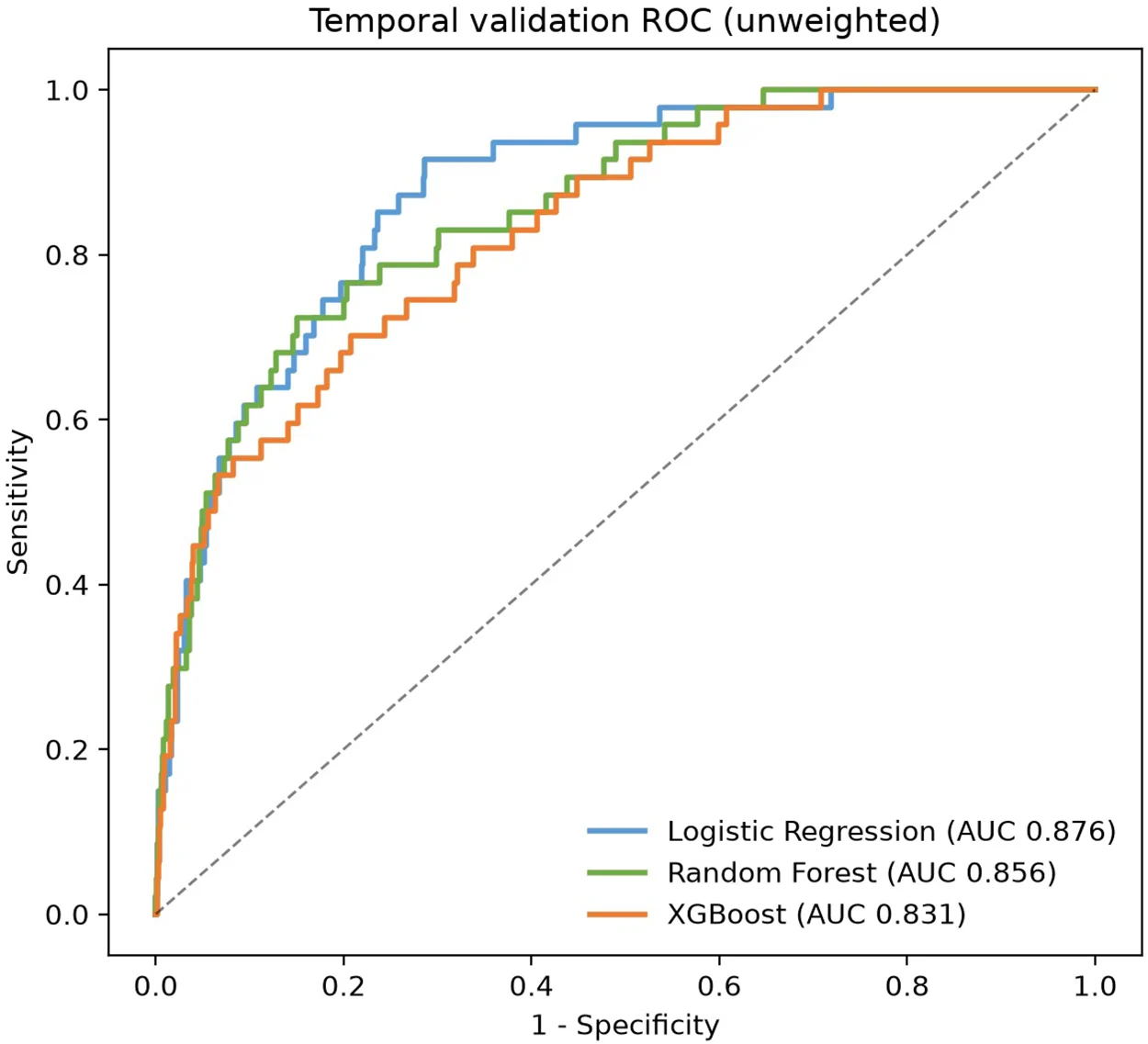
Receiver operating characteristic curves for logistic regression, random forest, and XGBoost evaluated on the temporal validation set (2017–2019; *n* = 2,210). AUC, area under the curve.

**Figure 3:**
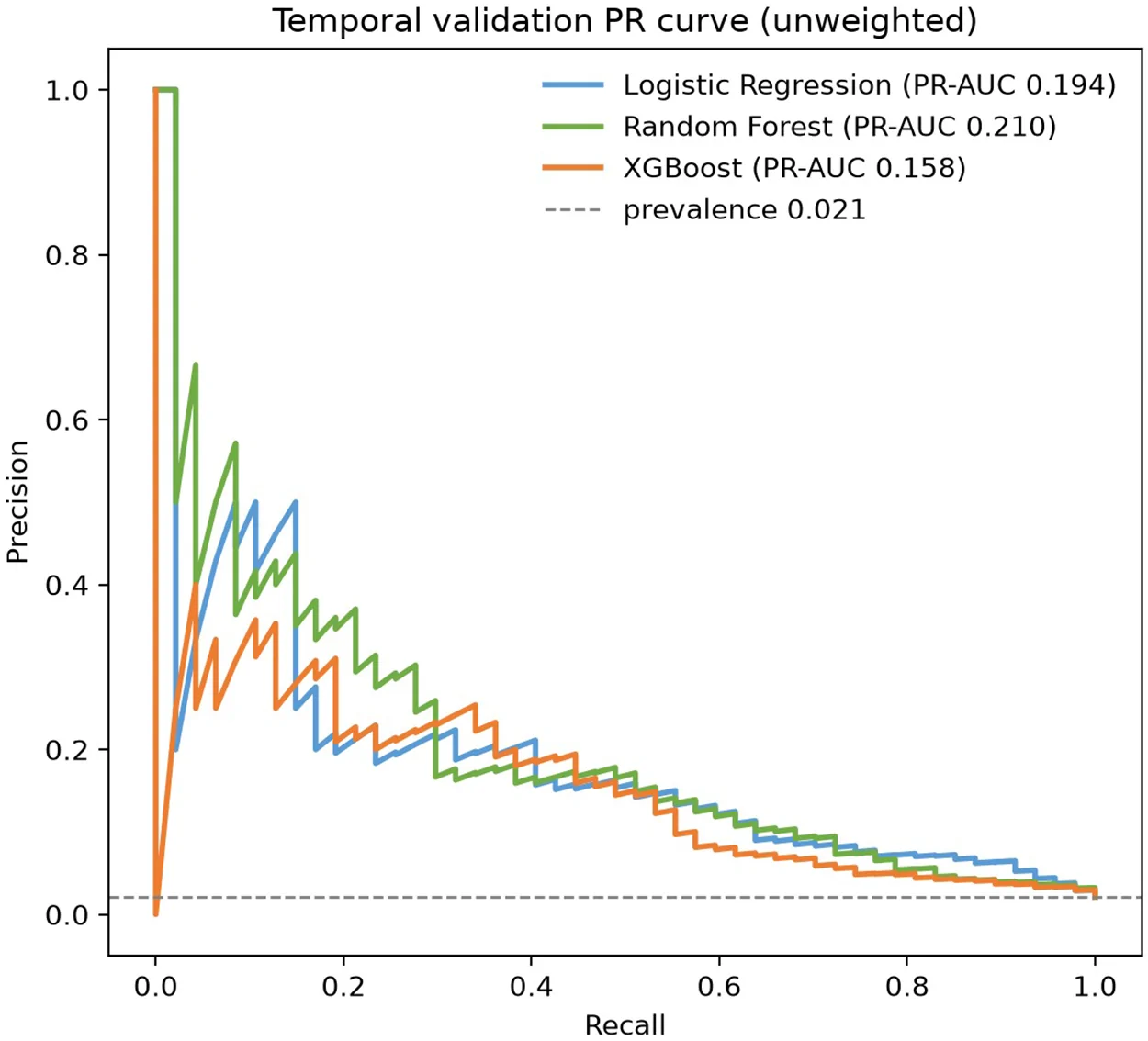
Precision-recall curves on the temporal validation set. The dashed horizontal line indicates baseline mortality prevalence (2.1%).

Under the unweighted, nested cross-validation comparison, logistic regression achieved the highest temporal AUC [0.876, 95% CI (0.828, 0.917)], followed by random forest [0.856, 95% CI (0.797, 0.907)] and XGBoost [0.831, 95% CI (0.772, 0.886)] (Table 2). Paired-bootstrap comparisons on the same validation cases showed small differences: *Δ*AUC for LR vs. RF was +0.020 [95% CI (−0.025, +0.061)] and for LR vs. XGBoost was +0.044 (95% CI (0.002, 0.084); the interval vs. XGBoost marginally excluded zero, whereas that vs. RF did not (Table 3). The temporal PR-AUC was 0.194 for LR, 0.210 for RF, and 0.158 for XGBoost; RF achieved the highest PR-AUC. ROC curves are presented in Figure 2 and PR curves in Figure 3.

**Table 2:** Model performance in internal cross-validation and temporal validation.

Model	Internal CV AUC	Temporal AUC (95% CI)	Temporal PR-AUC	Calibration slope
Logistic Regression	0.848	0.876 (0.828,0.917)	0.194	1.05
Random Forest	0.869	0.856 (0.797,0.907)	0.210	1.03
XGBoost	0.850	0.831 (0.772,0.886)	0.158	1.13

AUC, area under the receiver operating characteristic curve; CV, cross-validation; CI, confidence interval; *Δ*AUC, temporal AUC minus internal CV AUC; PR-AUC, area under the precision-recall curve. Internal AUC values and 95% CIs are means from 5-fold stratified cross-validation. Temporal AUC 95% confidence intervals were obtained by bootstrap (2,000 resamples); *Δ*AUC confidence intervals all included zero.

**Table 3:** Paired comparisons of discrimination on temporal validation.

Comparison	*Δ*AUC (95% CI)	*Δ*PR-AUC (95% CI)
Logistic regression vs. random forest	+0.020 (−0.025, +0.061)	−0.017 (−0.068, +0.031)
Logistic regression vs. XGBoost	+0.044 (+0.002, +0.084)	+0.031 (−0.038, +0.110)
Random forest vs. XGBoost	+0.024 (+0.002, +0.044)	+0.048 (−0.013, +0.119)

*Δ*, difference; CI, paired-bootstrap 95% confidence interval (2,000 resamples on the same validation cases). The *Δ*AUC intervals vs. XGBoost marginally exclude zero; all *Δ*PR-AUC intervals include zero.

Coefficients of the unweighted logistic model with bootstrap 95% confidence intervals are reported in Supplementary Table S2.

### 3.3 Model calibration

**Figure 4:**
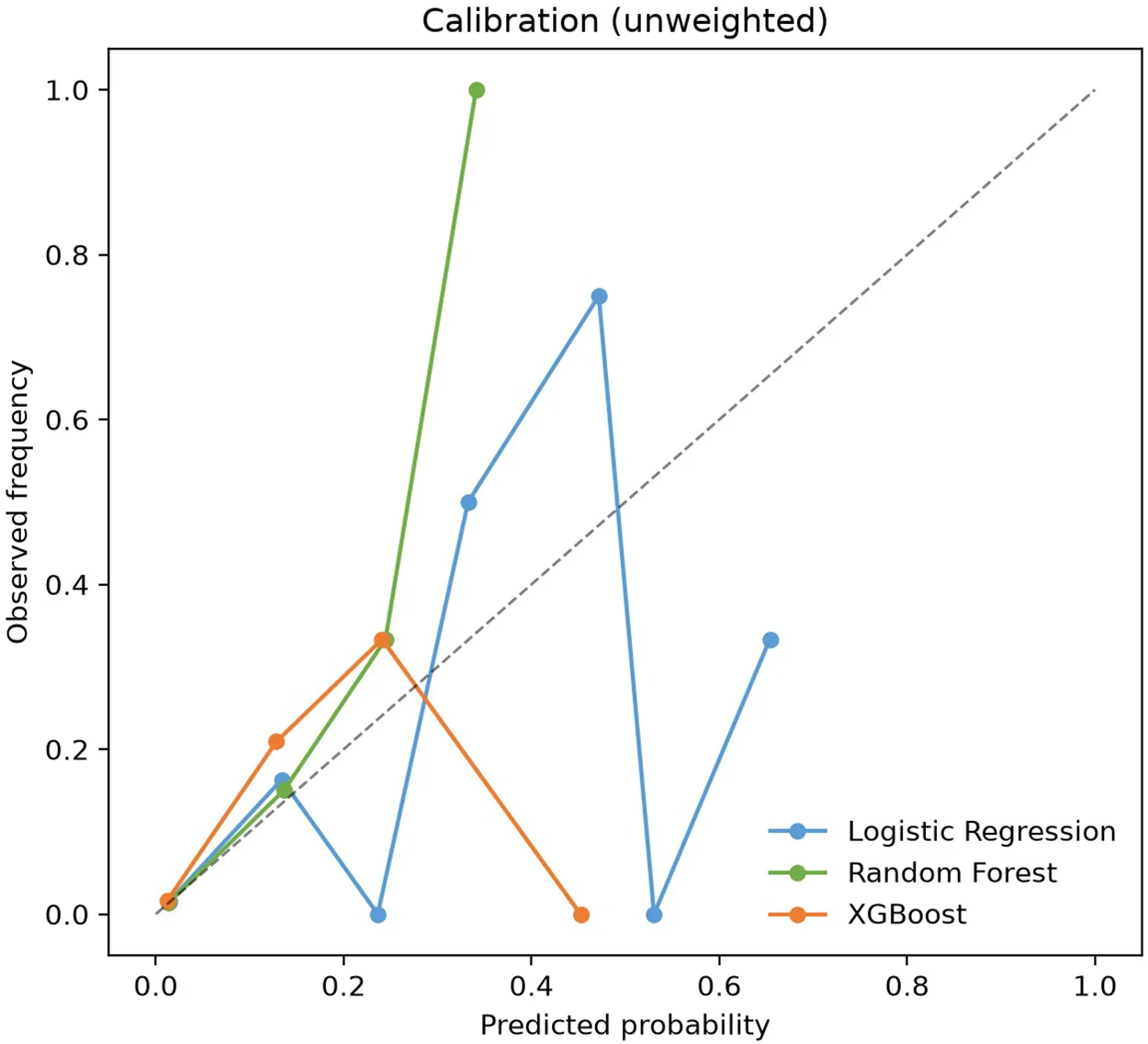
Calibration plots for the unweighted models on the temporal validation set. The dashed diagonal line represents perfect calibration. Brier scores are indicated in each panel.

Calibration plots for the temporal validation set are presented in Figure 4. Because the primary models were fit without class weighting, the raw predicted probabilities are directly interpretable as individual risks. On the temporal validation set the calibration intercepts were near zero (LR +0.19; RF +0.14; XGBoost +0.80), the Brier scores were 0.019–0.020, and the calibration slopes were 1.05 (LR), 1.03 (RF), and 1.13 (XGBoost), indicating well-calibrated individual risk estimates. In the weighted sensitivity variant, raw outputs were prevalence-shifted (intercepts approximately −1.4 to −3.7), but after isotonic recalibration fitted on development out-of-fold predictions the intercepts returned to near zero and the Brier scores fell to 0.019–0.020 (Supplementary Figure).

### 3.4 Feature importance and SHAP analysis

**Figure 5:**
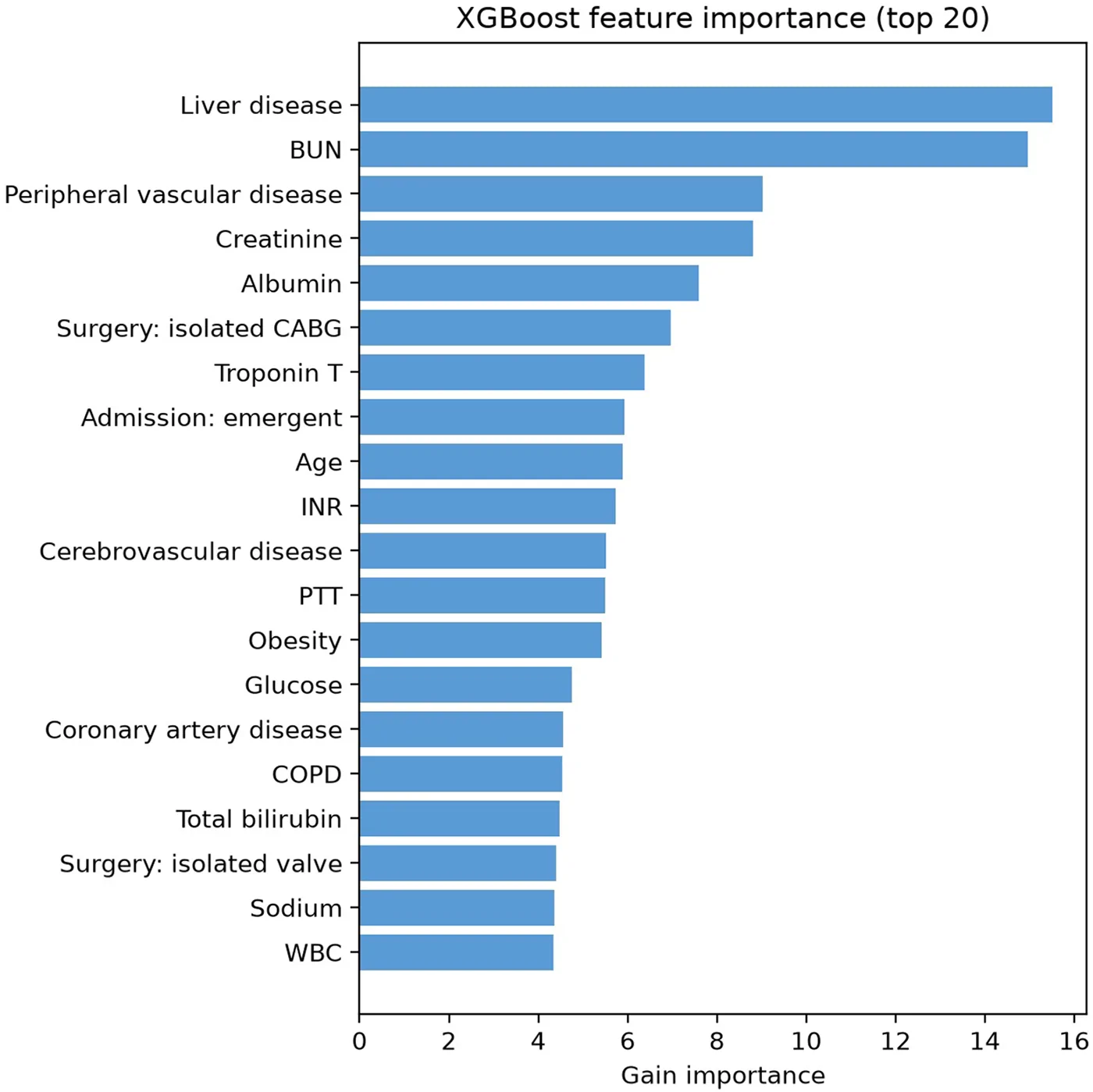
Top 20 feature importances from the XGBoost model, ranked by gain. BUN, blood urea nitrogen; CABG, coronary artery bypass grafting; CAD, coronary artery disease; COPD, chronic obstructive pulmonary disease; INR, international normalized ratio; PTT, partial thromboplastin time; WBC, white blood cell count.

**Figure 6:**
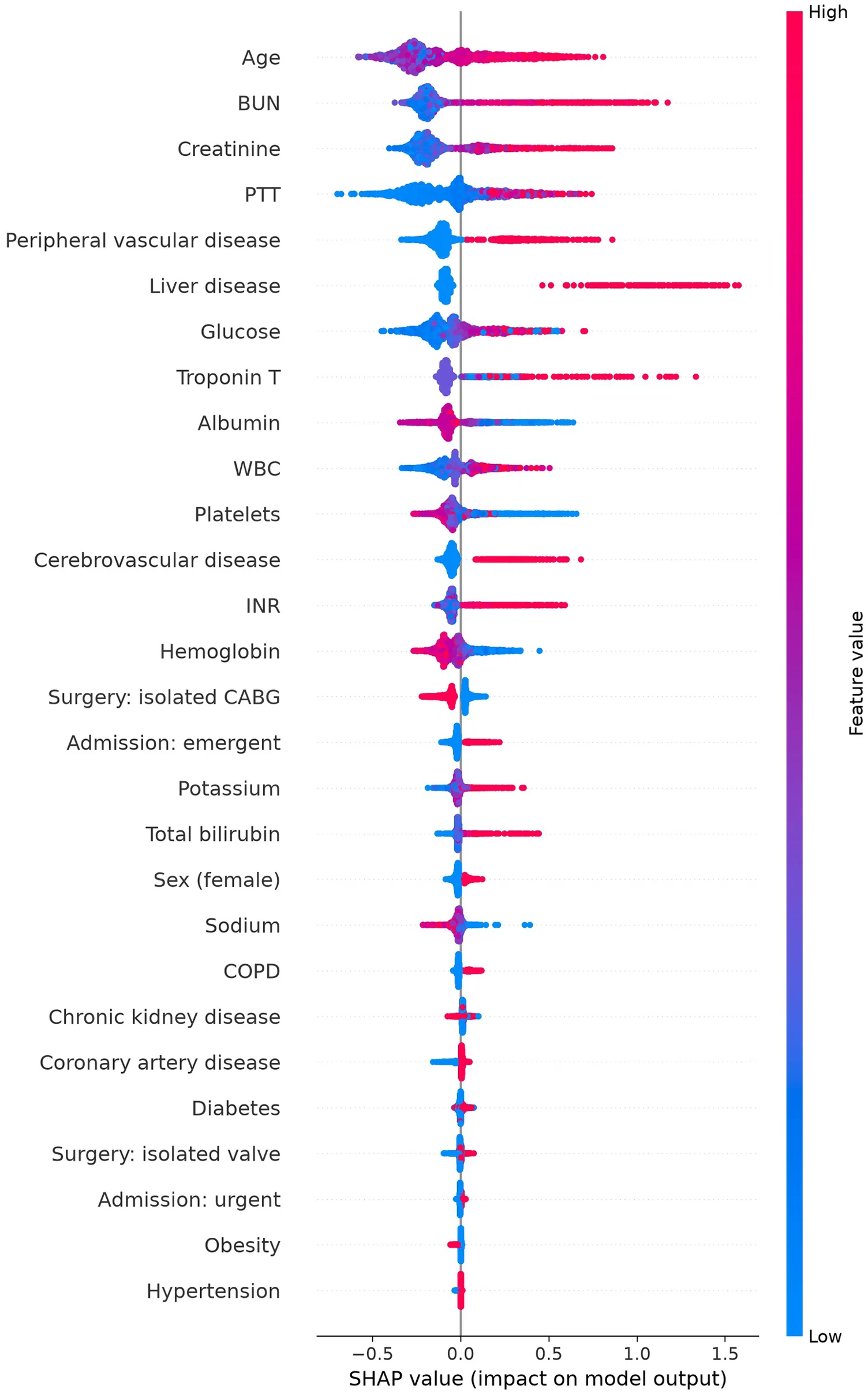
SHAP summary plot. Each point represents one patient. Red indicates higher values; blue indicates lower. Positive SHAP values indicate increased predicted mortality risk. BUN, blood urea nitrogen; CABG, coronary artery bypass grafting; CAD, coronary artery disease; INR, international normalized ratio; PTT, partial thromboplastin time; WBC, white blood cell count.

**Figure 7:**
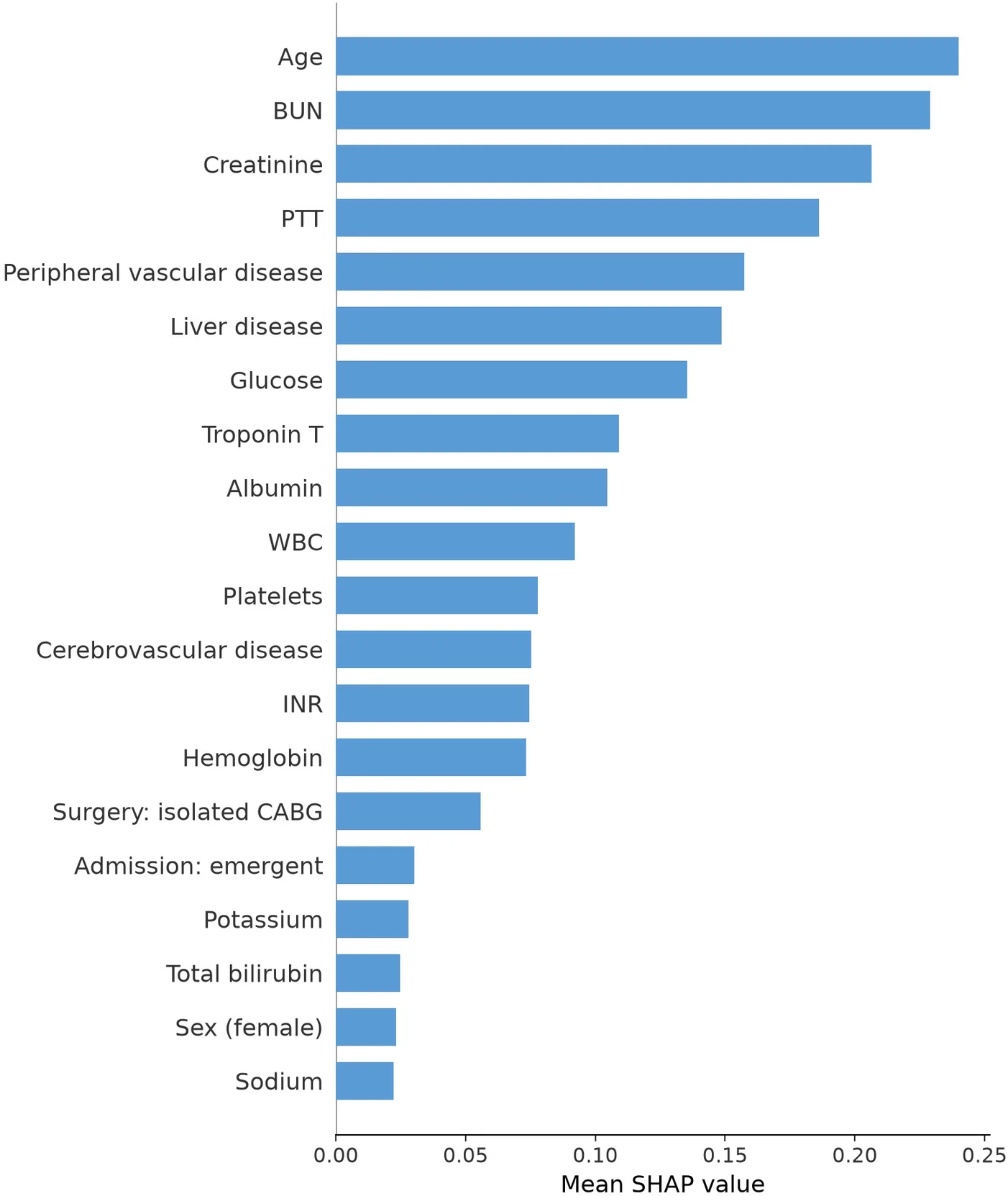
Mean absolute SHAP values for the top 20 features (*n* = 2,210). BUN, blood urea nitrogen; CABG, coronary artery bypass grafting; CAD, coronary artery disease; INR, international normalized ratio; PTT, partial thromboplastin time; WBC, white blood cell count.

XGBoost feature importance analysis (Figure 5) and SHAP analysis (Figures 6, 7) identified age, BUN, creatinine, PTT, glucose, and troponin T as the most influential continuous predictors, while peripheral vascular disease, liver disease, and cerebrovascular disease were the most influential comorbidities. Higher age, elevated BUN, creatinine, glucose, WBC count, and PTT were associated with increased predicted mortality risk, whereas higher hemoglobin, albumin, and platelet count were protective. Among comorbidities, peripheral vascular disease and liver disease showed the largest increases in predicted mortality risk (Figure 6).

## 4 Discussion

In this analysis of 9,956 cardiac surgery patients with temporal validation, logistic regression achieved the highest numerical temporal AUC (0.876), followed by random forest (0.856) and XGBoost (0.831); the between-model differences were small, with only the LR–XGBoost difference marginally excluding zero. Age, BUN, creatinine, and PTT were the key predictors of mortality.

The temporal stability of all three models was reasonable. Under the unweighted comparison, LR's temporal AUC (0.876) was slightly higher than its internal CV AUC (0.848), whereas RF and XGBoost showed small numerical declines (RF, from 0.869 to 0.856; XGBoost, from 0.850 to 0.831); none of these changes was statistically significant, and the paired differences in temporal discrimination were small. With only 163 deaths in the training set (2.1% prevalence), between-model differences in discrimination are difficult to establish with statistical confidence, and the present results should be interpreted as showing that a simple linear model performs no worse than more complex ensembles rather than that any approach is intrinsically more temporally stable.

In terms of calibration, the unweighted models were all well calibrated on the temporal validation set (calibration slopes 1.03–1.13, intercepts near zero, Brier score 0.019–0.020 for all three models). The strongly negative calibration intercepts that characterized earlier weighted analyses were an artifact of class weighting, which shifts raw probabilities toward a balanced prevalence; removing weighting returned the intercepts to near zero, confirming that the unweighted model provides interpretable individual probabilities. When class weighting is retained to aid ranking, recalibration on development out-of-fold predictions restores calibration (see Section 3).

The temporal AUC of 0.876 for LR is comparable to prior cardiac surgery ML studies. Allyn et al. reported an AUC of 0.795 (10), Benedetto et al. reported pooled c-statistics of 0.88 for machine learning versus 0.81 for logistic regression, a modest advantage whose clinical impact was considered uncertain (11), and Kilic et al. achieved an AUC of 0.808 using XGBoost (12). Critically, these studies predominantly used random splits, which can overestimate real-world performance (14). Our results illustrate this pitfall: RF and XGBoost achieved internal AUCs of 0.869 and 0.850, respectively, and both showed small numerical declines in temporal validation, underscoring the importance of time-based evaluation. It is important to emphasize, however, that this study compares the relative performance of three statistical modeling approaches rather than demonstrating superiority over established clinical risk scores such as EuroSCORE II or the STS score. Direct comparison with these instruments was not feasible, as key predictor variables required by these scores are not captured in MIMIC-IV (see Section 5).

SHAP analysis identified age, BUN, creatinine, and PTT as the dominant predictors. Renal function markers (BUN and creatinine) likely capture both chronic kidney disease severity and acute hemodynamic compromise (25), while PTT reflects coagulation status. Albumin, a marker of nutritional status and chronic inflammation, also contributed (26–28). The modest contribution of binary comorbidities suggests that continuous laboratory values capture more granular prognostic information. From a clinical perspective, an interpretable LR model offers a transparent and interpretable tool for risk stratification, whereas tree-based ensembles require *post-hoc* explanation methods.

These findings contribute to a growing body of evidence that the advantages of complex ML models over logistic regression are often modest in low-event clinical prediction settings (29, 30). While the present study demonstrates that a simple linear model performed no worse than tree-based ensembles under temporal validation within a single institution, it should be noted that temporal validation within one center does not replace external validation across different healthcare systems and patient populations. The magnitude and pattern of between-model differences observed here may differ when models are transported to institutions with different case mixes, practice patterns, and coding conventions. Nonetheless, the finding that logistic regression matched or exceeded the tree-based models while offering calibrated individual probabilities supports the emerging consensus that rigorous temporal or external validation should be a prerequisite for reporting ML model performance in clinical prediction studies (17).

Several directions for future research emerge from this work. External validation using multi-institutional registries, such as the STS database, would be valuable to confirm the generalizability of our findings. Incorporating unstructured clinical data through natural language processing of echocardiogram reports and operative notes could address the limitation of missing key prognostic variables. Prospective evaluation of an LR-based risk calculator in a clinical setting would provide evidence regarding real-world utility and clinician acceptance.

## 5 Limitations

Several limitations warrant consideration. The MIMIC-IV database reflects a single US academic medical center; external validation in independent cohorts is needed. Direct comparison with EuroSCORE II or STS score was not feasible because required variables—left ventricular ejection fraction, pulmonary artery pressure, and detailed operative urgency—are incompletely captured in MIMIC-IV. Consequently, this study compares the relative performance of three statistical modeling approaches and should not be interpreted as demonstrating superiority over established clinical risk prediction tools. Several established prognostic factors in cardiac surgery, including left ventricular ejection fraction, New York Heart Association functional class, cardiopulmonary bypass time, and intraoperative transfusion volume, were unavailable in the data extracted from MIMIC-IV, which may have limited model discrimination. The low event rate (2.1%) limits statistical power. Furthermore, several laboratory variables had substantial missing data (albumin 64%, troponin T 84%), and median imputation—while transparent and computationally straightforward—may have attenuated variance and weakened predictor-outcome associations, potentially biasing model calibration and discrimination estimates. ICD-code-based comorbidity ascertainment may be subject to misclassification, and differential coding practices across time could influence temporal performance. Patients from 2020 to 2022 were excluded, so findings may not extend to the peri-pandemic population. The MIMIC-IV cohort may not represent cardiac surgery populations in other healthcare systems. Although temporal validation provides a more rigorous assessment than random data splitting, it does not replace independent external validation across different healthcare systems, geographic regions, and patient populations. Sociodemographic subgroup differences in performance were not examined because MIMIC-IV captures only age and sex as demographic variables. When deployed, missing laboratory values would be replaced by the development-set medians reported in the analysis repository, and out-of-range values should be flagged for review; the model requires no patient interaction and is intended for interpretation by trained clinicians.

## 6 Conclusion

In this temporal validation study of 9,956 cardiac surgery patients, logistic regression achieved the highest numerical discrimination for in-hospital mortality (temporal AUC 0.876), but the advantage over random forest (0.856) and XGBoost (0.831) was small, with only the difference vs. XGBoost marginally excluding zero. Age, BUN, creatinine, and PTT were the predominant predictors. Within this single-center cohort, an interpretable logistic regression model matched or exceeded the complex ML algorithms while providing well-calibrated individual probability estimates, but these findings do not establish that any single approach should be preferred for early-admission surgical risk stratification when event rates are low. External validation in multi-institutional cohorts is needed to confirm the generalizability of these results.

## Data Availability

Publicly available datasets were analyzed in this study. This data can be found here: https://physionet.org/content/mimiciv/3.1/.

## References

[1] Nashef SAM Roques F Sharples LD Nilsson J Smith C Goldstone AR. EuroSCORE II. Eur J Cardiothorac Surg. (2012) 41(4):734–45. 10.1093/ejcts/ezs043 22378855

[2] Shahian DM O'Brien SM Filardo G Ferraris VA Haan CK Rich JB. The society of thoracic surgeons 2008 cardiac surgery risk models: part 1—coronary artery bypass grafting surgery. Ann Thorac Surg. (2009) 88(1 Suppl):S2–S22. 10.1016/j.athoracsur.2009.05.053 19559822

[3] Bowdish ME D'Agostino RS Thourani VH Schwann TA Krohn C Desai N. STS adult cardiac surgery database: 2021 update on outcomes, quality, and research. Ann Thorac Surg. (2021) 111(6):1770–80. 10.1016/j.athoracsur.2021.03.043 33794156

[4] Hickey GL Grant SW Murphy GJ Bhabra M Pagano D McAllister K. Dynamic trends in cardiac surgery: why the logistic EuroSCORE is no longer suitable for contemporary cardiac surgery. Eur J Cardiothorac Surg. (2013) 43(6):1146–52. 10.1093/ejcts/ezs584 23152436 PMC3655624

[5] Siregar S Groenwold RHH De Heer F Bots ML Van Der Graaf Y Van Herwerden LA. Performance of the original EuroSCORE. Eur J Cardiothorac Surg. (2012) 41(4):746–54. 10.1093/ejcts/ezr285 22290922

[6] Barili F Pacini D Capo A Rasovic O Grossi C Alamanni F. Does EuroSCORE II perform better than its original versions? A multicentre validation study. Eur Heart J. (2013) 34(1):22–9. 10.1093/eurheartj/ehs342 23028171

[7] Howell NJ Head SJ Freemantle N Van Der Meulen TA Senanayake E Menon A. The new EuroSCORE II does not improve prediction of mortality in high-risk patients. Eur J Cardiothorac Surg. (2013) 44(6):1009–15. 10.1093/ejcts/ezt174 23536616

[8] Breiman L. Random forests. Mach Learn. (2001) 45(1):5–32. 10.1023/A:1010933404324

[9] Chen T Guestrin C. XGBoost: a scalable tree boosting system. In: Proceedings of the 22nd ACM SIGKDD International Conference on Knowledge Discovery and Data Mining (2016), 785–94

[10] Allyn J Allou N Augustin P Philip I Martinet O Belghiti M. A comparison of a machine learning model with EuroSCORE II in predicting mortality after elective cardiac surgery: a decision curve analysis. PLoS One. (2017) 12(1):e0169772. 10.1371/journal.pone.0169772 28060903 PMC5218502

[11] Benedetto U Dimagli A Sinha S Cocomello L Gibbison B Caputo M. Machine learning improves mortality risk prediction after cardiac surgery. J Thorac Cardiovasc Surg. (2022) 163(6):2075–87.e9. 10.1016/j.jtcvs.2020.07.105 32900480

[12] Kilic A Goyal A Miller JK Gjekmarkaj E Tam WL Gleason TG. Predictive utility of a machine learning algorithm in estimating mortality risk in cardiac surgery. Ann Thorac Surg. (2020) 109(6):1811–9. 10.1016/j.athoracsur.2019.09.049 31706872

[13] Christodoulou E Ma J Collins GS Steyerberg EW Verbakel JY Van Calster B. A systematic review shows no performance benefit of machine learning over logistic regression for clinical prediction models. J Clin Epidemiol. (2019) 110:12–22. 10.1016/j.jclinepi.2019.02.004 30763612

[14] Gravesteijn BY Nieboer D Ercole A Lingsma HF Nelson D Van Calster B. Machine learning algorithms performed no better than regression models for prognostication in traumatic brain injury. J Clin Epidemiol. (2020) 122:95–107. 10.1016/j.jclinepi.2020.03.005 32201256

[15] Davis SE Lasko TA Chen G Siew ED Matheny ME. Calibration drift in regression and machine learning models for acute kidney injury. J Am Med Inform Assoc. (2017) 24(6):1052–61. 10.1093/jamia/ocx030 28379439 PMC6080675

[16] Nestor B McDermott MBA Boag W Berner G Naumann T Hughes MC. Feature robustness in non-stationary health records: caveats to deployable model performance in common clinical machine learning tasks. Proc Mach Learn Res. (2019) 106:381–405.

[17] Collins GS Moons KGM. Reporting of artificial intelligence prediction models. Lancet. (2019) 393(10181):1577–9. 10.1016/S0140-6736(19)30037-6 31007185

[18] Johnson AEW Bulgarelli L Shen L Gayles A Shammout A Horng S. MIMIC-IV, a freely accessible electronic health record dataset. Sci Data. (2023) 10(1):1. 10.1038/s41597-022-01899-x 36596836 PMC9810617

[19] Collins GS Moons KGM Dhiman P Riley RD Beam AL Van Calster B. TRIPOD + AI statement: updated guidance for reporting clinical prediction models that use regression or machine learning methods. Br Med J. (2024) 385:e078378. 10.1136/bmj-2023-078378 38626948 PMC11019967

[20] Lundberg SM Lee SI. A unified approach to interpreting model predictions. In: Guyon I von Luxburg U Bengio S Wallach H Fergus R Vishwanathan S. editors. Advances in Neural Information Processing Systems. Red Hook: Curran Associates, Inc. (2017). p. 4765–74.

[21] Van Rossum G Drake FL. Python 3 Reference Manual. Scotts Valley, CA: CreateSpace (2009).

[22] Pedregosa F Varoquaux G Gramfort A Michel V Thirion B Grisel O. Scikit-learn: machine learning in Python. J Mach Learn Res. (2011) 12:2825–30.

[23] Steyerberg EW Vickers AJ Cook NR Gerds T Gonen M Obuchowski N. Assessing the performance of prediction models: a framework for traditional and novel measures. Epidemiology. (2010) 21(1):128–38. 10.1097/EDE.0b013e3181c30fb2 20010215 PMC3575184

[24] Berkelmans GFN Read SH Gudbjörnsdottir S Wild SH Franzen S Van Der Graaf Y. Population median imputation was noninferior to complex approaches for imputing missing values in cardiovascular prediction models in clinical practice. J Clin Epidemiol. (2022) 145:70–80. 10.1016/j.jclinepi.2022.01.011 35066115

[25] Thakar CV Arrigain S Worley S Yared JP Paganini EP. A clinical score to predict acute renal failure after cardiac surgery. J Am Soc Nephrol. (2005) 16(1):162–8. 10.1681/ASN.2004040331 15563569

[26] Bhamidipati CM LaPar DJ Mehta GS Kern JA Upchurch GR Kron IL. Albumin is a better predictor of outcomes than body mass index following coronary artery bypass grafting. Ann Thorac Surg. (2011) 91(5):1502–8. 10.1016/j.surg.2011.07.056 22000173 PMC3204902

[27] de La Cruz KI Bakaeen FG Wang XL Huh J LeMaire SA Coselli JS. Hypoalbuminemia and long-term survival after coronary artery bypass. Ann Thorac Surg. (2011) 91(3):762–7. 10.1016/j.athoracsur.2010.09.004 21352977

[28] Engelman DT Adams DH Byrne JG Aranki SF Collins JJ Couper GS. Impact of body mass index and albumin on morbidity and mortality after cardiac surgery. J Thorac Cardiovasc Surg. (1999) 118(5):866–73. 10.1016/S0022-5223(99)70056-5 10534692

[29] Finlayson SG Subbaswamy A Singh K Bowers J Kupke A Zittrain J. The clinician and dataset shift in artificial intelligence. N Engl J Med. (2021) 385(3):283–6. 10.1056/NEJMc2104626 34260843 PMC8665481

[30] Beam AL Kohane IS. Big data and machine learning in health care. JAMA. (2018) 319(13):1317–8. 10.1001/jama.2017.18391 29532063

[31] Murphy AH. A new vector partition of the probability score. J Appl Meteorol. (1973) 12(4):595–600. 10.1175/1520-0450(1973)012<0595:ANVPOT>2.0.CO;2

