# Supplementary material for: Predicting in-hospital mortality after cardiac surgery using machine learning: temporal validation in the MIMIC-IV database
Source: Front Cardiovasc Med. 2026 Aug 31;13:1914633. doi: 10.3389/fcvm.2026.1914633 (PMC13572127; doi:10.3389/fcvm.2026.1914633)
Supplement: Supplementary file 1 [file Table1.docx]

**Supplementary Table S1**

Laboratory values and missing rates for all 13 laboratory variables

| **Variable** | **Train available (n)** | **Train missing (%)** | **Train mean ± SD** | **Validation available (n)** | **Validation missing (%)** | **Validation mean ± SD** | **p-value** |
| --- | --- | --- | --- | --- | --- | --- | --- |
| Creatinine, mg/dL | 7422 | 4.2 | 1.2 ± 1.1 | 2108 | 4.6 | 1.1 ± 1.0 | 0.153 |
| Hemoglobin, g/dL | 7413 | 4.3 | 12.2 ± 2.1 | 2134 | 3.4 | 12.3 ± 2.0 | 0.004 |
| WBC count, ×10⁹/L | 7335 | 5.3 | 10.2 ± 5.8 | 2115 | 4.3 | 10.1 ± 4.7 | 0.559 |
| Platelets, ×10⁹/L | 7378 | 4.8 | 188.6 ± 72.7 | 2118 | 4.2 | 182.2 ± 69.8 | 0.000 |
| Sodium, mmol/L | 7006 | 9.6 | 139.2 ± 3.2 | 1559 | 29.5 | 139.6 ± 3.3 | 0.000 |
| Potassium, mmol/L | 7248 | 6.4 | 4.2 ± 0.5 | 1928 | 12.8 | 4.3 ± 0.5 | 0.000 |
| Glucose, mg/dL | 7418 | 4.2 | 135.4 ± 50.4 | 2124 | 3.9 | 136.3 ± 55.8 | 0.506 |
| Blood urea nitrogen, mg/dL | 7414 | 4.3 | 21.4 ± 13.0 | 2108 | 4.6 | 20.0 ± 11.4 | 0.000 |
| Albumin, g/dL | 2754 | 64.4 | 3.9 ± 0.5 | 889 | 59.8 | 3.9 ± 0.5 | 0.101 |
| Total bilirubin, mg/dL | 3209 | 58.6 | 0.7 ± 0.6 | 1062 | 51.9 | 0.6 ± 0.4 | 0.380 |
| International normalized ratio | 6994 | 9.7 | 1.3 ± 0.4 | 2050 | 7.2 | 1.3 ± 0.4 | 0.408 |
| Partial thromboplastin time, sec | 7008 | 9.5 | 39.5 ± 23.9 | 2038 | 7.8 | 41.5 ± 27.5 | 0.002 |
| Troponin T, ng/mL | 1208 | 84.4 | 0.8 ± 1.9 | 327 | 85.2 | 0.8 ± 1.7 | 0.676 |

*Values are presented as mean ± standard deviation. Training set: n = 7,746. Validation set: n = 2,210. Laboratory values were obtained within 24 hours of admission. Missing rates reflect the proportion of patients without an available measurement. p-values were calculated using the independent t-test for available values.*
