# Supplementary material for: Predicting in-hospital mortality after cardiac surgery using machine learning: temporal validation in the MIMIC-IV database
Source: Front Cardiovasc Med. 2026 Aug 31;13:1914633. doi: 10.3389/fcvm.2026.1914633 (PMC13572127; doi:10.3389/fcvm.2026.1914633)
Supplement: Supplementary file 2 [file Table2.docx]

**Supplementary Table S2**

Coefficients of the unweighted logistic model

| **Predictor** | **Coefficient** | **OR** | **95% CI (bootstrap)** |
| --- | --- | --- | --- |
| Age | 0.433 | 1.54* | 1.24–2.03 |
| Sex (female) | 0.095 | 1.10 | 0.91–1.32 |
| Admission type: emergent | 0.365 | 1.44* | 1.20–1.80 |
| Admission type: urgent | 0.248 | 1.28* | 1.04–1.61 |
| Surgery type: isolated CABG | -0.335 | 0.72* | 0.55–0.90 |
| Surgery type: isolated valve | 0.030 | 1.03 | 0.80–1.30 |
| Hypertension | 0.088 | 1.09 | 0.90–1.39 |
| Diabetes mellitus | 0.029 | 1.03 | 0.83–1.27 |
| Chronic kidney disease | 0.040 | 1.04 | 0.86–1.24 |
| COPD | 0.131 | 1.14 | 0.98–1.34 |
| Coronary artery disease | 0.212 | 1.24 | 0.98–1.59 |
| Cerebrovascular disease | 0.219 | 1.24* | 1.10–1.39 |
| Peripheral vascular disease | 0.325 | 1.38* | 1.20–1.60 |
| Liver disease | 0.311 | 1.37* | 1.24–1.52 |
| Obesity | -0.190 | 0.83* | 0.65–0.998 |
| Creatinine | 0.163 | 1.18* | 1.04–1.33 |
| Hemoglobin | -0.064 | 0.94 | 0.75–1.09 |
| White blood cell count | 0.057 | 1.06 | 0.95–1.20 |
| Platelet count | -0.079 | 0.92 | 0.76–1.08 |
| Sodium | -0.070 | 0.93 | 0.80–1.10 |
| Potassium | 0.195 | 1.21* | 1.02–1.42 |
| Glucose | 0.159 | 1.17 | 1.000–1.36 |
| Blood urea nitrogen | 0.162 | 1.18* | 1.02–1.36 |
| Albumin | -0.210 | 0.81* | 0.71–0.93 |
| Total bilirubin | 0.098 | 1.10 | 0.98–1.25 |
| INR | 0.096 | 1.10 | 0.99–1.19 |
| PTT | 0.187 | 1.21* | 1.06–1.33 |
| Troponin T | 0.079 | 1.08* | 1.01–1.17 |

*Coefficients are per 1 SD of continuous predictors (or per unit for binary indicators); OR, odds ratio = exp(coefficient); CI, bootstrap 95% confidence interval (1,000 resamples, repeating imputation and scaling within each resample). *95% CI excludes 1. No p-values are reported; coefficients are shown for model transparency, not as independently validated prognostic factors. Category coding: sex (1 = female, 0 = male); admission type (reference = elective; indicator variables for urgent and emergent); surgery type (reference = combined CABG + valve; indicator variables for isolated CABG and isolated valve); comorbidities and other binary indicators coded 0/1 (1 = present).*
