# Supplementary material for: Predicting in-hospital mortality after cardiac surgery using machine learning: temporal validation in the MIMIC-IV database
Source: Front Cardiovasc Med. 2026 Aug 31;13:1914633. doi: 10.3389/fcvm.2026.1914633 (PMC13572127; doi:10.3389/fcvm.2026.1914633)
Supplement: Supplementary file 3 [file Table3.docx]

**Supplementary Table S3**

Missing-data sensitivity analyses (temporal AUC, unweighted models)

| **Scenario** | **Logistic regression** | **Random forest** | **XGBoost** |
| --- | --- | --- | --- |
| Main analysis (median imputation) | 0.876 | 0.859 | 0.839 |
| Drop >50%-missing variables (albumin, total bilirubin, troponin T) | 0.869 | 0.849 | 0.830 |
| Median imputation + missingness indicators | 0.870 | 0.852 | 0.834 |
| Multivariate iterative (MICE-style) imputation | 0.881 | 0.847 | 0.842 |

*The main-analysis row repeats the unweighted temporal AUC from Table 2. Each scenario reruns the full nested cross-validation comparison.*
