# Supplementary material for: Predicting in-hospital mortality after cardiac surgery using machine learning: temporal validation in the MIMIC-IV database
Source: Front Cardiovasc Med. 2026 Aug 31;13:1914633. doi: 10.3389/fcvm.2026.1914633 (PMC13572127; doi:10.3389/fcvm.2026.1914633)
Supplement: Supplementary file 4 [file Table4.docx]

**Supplementary Table S4**

Temporal AUC stratified by admission type (unweighted models, temporal validation)

| **Admission type** | **n (events)** | **Logistic regression** | **Random forest** | **XGBoost** |
| --- | --- | --- | --- | --- |
| Elective | 1054 (8) | 0.81 | 0.81 | 0.76 |
| Urgent | 974 (26) | 0.88 | 0.87 | 0.84 |
| Emergent | 182 (13) | 0.76 | 0.73 | 0.76 |

*Strata are small and these AUCs are unstable; they are reported for interpretation rather than inference. The three models performed similarly within every stratum, with no consistent logistic-regression advantage (XGBoost was numerically highest in the emergent stratum).*
