# Supplementary material for: Predicting in-hospital mortality after cardiac surgery using machine learning: temporal validation in the MIMIC-IV database
Source: Front Cardiovasc Med. 2026 Aug 31;13:1914633. doi: 10.3389/fcvm.2026.1914633 (PMC13572127; doi:10.3389/fcvm.2026.1914633)
Supplement: Supplementary file 5 [file Table5.docx]

# TRIPOD+AI Checklist — Completion Table

**Manuscript:** Predicting In-Hospital Mortality After Cardiac Surgery Using Machine Learning: Temporal Validation in the MIMIC-IV Database

**Checklist reference:** Collins GS, Moons KGM, Dhiman P, Riley RD, Beam AL, Van Calster B, et al. TRIPOD+AI statement: updated guidance for reporting clinical prediction models that use regression or machine learning methods. *BMJ*. 2024;385:e078378. doi:10.1136/bmj-2023-078378.

**D** = item relevant only to *development* of a prediction model; **E** = item relevant only to *evaluation*; **D;E** = both. “Location” gives the section of the revised manuscript (R3). Items marked “Not applicable” are explained in the Notes at the end.

| Section / Topic | Item | D/E | Checklist item | Location in manuscript |
| --- | --- | --- | --- | --- |
| **TITLE** |  |  |  |  |
| Title | 1 | D;E | Identify the study as developing or evaluating the performance of a multivariable prediction model, the target population, and the outcome to be predicted | Title |
| **ABSTRACT** |  |  |  |  |
| Abstract | 2 | D;E | See TRIPOD+AI for Abstracts checklist | Abstract (structured) |
| **INTRODUCTION** |  |  |  |  |
| Background | 3a | D;E | Explain the healthcare context (diagnostic or prognostic) and rationale for developing/evaluating the model, including references to existing models | Introduction ¶1–2 |
|  | 3b | D;E | Describe the target population and intended purpose of the model in the context of the care pathway, including its intended users | Introduction ¶2; §2.2 |
|  | 3c | D;E | Describe any known health inequalities between sociodemographic groups | Limitations |
| Objectives | 4 | D;E | Specify the study objectives, including whether development or validation (or both) | Introduction ¶4 |
| **METHODS** |  |  |  |  |
| Data | 5a | D;E | Describe sources of data separately for development and evaluation datasets, the rationale for using these data, and representativeness | §2.1 |
|  | 5b | D;E | Specify dates of collected participant data, including start/end of accrual; if applicable, end of follow-up | §2.1 (2008–2019) |
| Participants | 6a | D;E | Specify key elements of the study setting, including number and location of centres | §2.1 (single centre, BIDMC) |
|  | 6b | D;E | Describe eligibility criteria for study participants | §2.1 |
|  | 6c | D;E | Give details of any treatments received, and how handled during model development/evaluation | §2.1–2.2 (CABG/valve) |
| Data preparation | 7 | D;E | Describe data pre-processing and quality checking, including whether similar across sociodemographic groups | §2.3 |
| Outcome | 8a | D;E | Define the outcome and time horizon, how/when assessed, rationale, and consistency across groups | §2.2 (in-hospital mortality) |
|  | 8b | D;E | If outcome assessment requires subjective interpretation, describe assessors’ qualifications/demographics | Not applicable (objective outcome) |
|  | 8c | D;E | Report actions to blind assessment of the outcome | Not applicable (objective outcome) |
| Predictors | 9a | D | Describe choice of initial predictors and any pre-selection before model building | §2.2 (all 29, no variable selection) |
|  | 9b | D;E | Clearly define all predictors, including how and when measured (and blinding) | §2.2 (within 24 h of admission) |
|  | 9c | D;E | If predictor measurement requires subjective interpretation, describe assessors’ qualifications/demographics | Not applicable (objective measurements) |
| Sample size | 10 | D;E | Explain how the study size was arrived at and justify sufficiency; include any sample size calculation | Limitations (all eligible patients; no formal calculation) |
| Missing data | 11 | D;E | Describe how missing data were handled; give reasons for omitting any data | §2.4 |
| Analytical methods | 12a | D | Describe how data were used (development/evaluation), including partitioning and sample size | §2.3 (temporal split) |
|  | 12b | D | Describe how predictors were handled (functional form, rescaling, transformation, standardisation) | §2.3 (median imputation, z-score) |
|  | 12c | D | Specify type of model, rationale, all model-building steps, hyperparameter tuning, internal validation | §2.3 (LR/RF/XGB, nested CV) |
|  | 12d | D;E | Describe if/how heterogeneity in parameter values and performance across clusters was handled and quantified | Not applicable (single centre); stratified by admission type, §3.2 & S4 |
|  | 12e | D;E | Specify all measures and plots used to evaluate performance and compare models | §2.4 |
|  | 12f | E | Describe any model updating (recalibration) arising from the evaluation | §2.3, §3.3 (isotonic recalibration) |
|  | 12g | E | Describe how model predictions were calculated (formula, code, object, API) | Data Availability (GitHub) |
| Class imbalance | 13 | D;E | If class imbalance methods were used, state why and how, and any recalibration | §2.3 (unweighted primary; weighted + recalibrated sensitivity) |
| Fairness | 14 | D;E | Describe any approaches used to address model fairness and their rationale | Limitations (case-mix shift); stratified performance S4 (see Notes) |
| Model output | 15 | D | Specify the output of the model (probabilities, classification) and rationale for any thresholds | §2.2 (probabilities; no classification) |
| Training vs evaluation | 16 | D;E | Identify any differences between development and evaluation data in setting, eligibility, outcome, predictors | §2.3; Table 1 |
| Ethical approval | 17 | D;E | Name the IRB/ethics committee and describe consent or waiver | Ethics Statement |
| **OPEN SCIENCE** |  |  |  |  |
| Funding | 18a | D;E | Give the source of funding and the role of the funders | Funding |
| Conflicts of interest | 18b | D;E | Declare any conflicts of interest and financial disclosures | Conflict of Interest |
| Protocol | 18c | D;E | Indicate where the study protocol can be accessed or state that it was not prepared | Ethics Statement |
| Registration | 18d | D;E | Provide registration information, or state that the study was not registered | Ethics Statement |
| Data sharing | 18e | D;E | Provide details of the availability of the study data | Data Availability (PhysioNet) |
| Code sharing | 18f | D;E | Provide details of the availability of the analytical code | Data Availability (GitHub) |
| **PATIENT & PUBLIC INVOLVEMENT** |  |  |  |  |
| Patient & Public Involvement | 19 | D;E | Provide details of any PPI during design/conduct/reporting, or state no involvement | Ethics Statement |
| **RESULTS** |  |  |  |  |
| Participants | 20a | D;E | Describe the flow of participants (with/without outcome, follow-up); a diagram may help | Figure 1 |
|  | 20b | D;E | Report characteristics overall and per setting, including key predictors, treatments, sample size, outcome events, missing data | Table 1 |
|  | 20c | E | For evaluation, show comparison with development data of the distribution of important predictors | Table 1 (training vs validation) |
| Model development | 21 | D;E | Specify the number of participants and outcome events in each analysis | §3.1 |
| Model specification | 22 | D | Provide details of the full prediction model (formula, code, object, API) and any restrictions | §2.3; Supplementary Table S2; GitHub |
| Model performance | 23a | D;E | Report performance estimates with confidence intervals, including key subgroups | Table 2, Table 3, S4 |
|  | 23b | D;E | If examined, report results of any heterogeneity in performance across clusters | Not applicable (single centre) |
| Model updating | 24 | E | Report results from any model updating, including updated model and performance | §3.3 |
| **DISCUSSION** |  |  |  |  |
| Interpretation | 25 | D;E | Give an overall interpretation of results, including fairness, in context of objectives and previous studies | Discussion |
| Limitations | 26 | D;E | Discuss limitations and their effects on biases, statistical uncertainty, and generalizability | Limitations |
| Usability of the model in current care | 27a | D | Describe how poor-quality or unavailable input data should be assessed and handled when implementing | Limitations |
|  | 27b | D | Specify whether users interact with the input data or model, and the level of expertise required | Limitations |
|  | 27c | D;E | Discuss next steps for future research, with a view to applicability and generalizability | Discussion |

## Notes

Items flagged “Not applicable” and the handling:

- **12d / 23b (clusters).** Single-centre retrospective cohort, so no between-cluster heterogeneity; heterogeneity was instead probed by admission-type strata (S4).
- **14 (fairness).** No explicit fairness algorithm was applied; the temporal case-mix shift (emergent 21.2% → 8.2%, urgent 31.9% → 44.1%) is discussed as a source of bias, and performance is additionally reported stratified by admission type.
- **3c / 18c / 18d / 19 / 27a / 27b.** Addressed by sentences added to the Ethics Statement and Limitations: sociodemographic subgroup differences were not examined (MIMIC-IV records only age and sex); no protocol was prepared; the study was not registered; there was no patient or public involvement; missing laboratory values would be replaced by development-set medians at deployment, with out-of-range values flagged for review; the model requires no patient interaction and is intended for trained clinicians.
